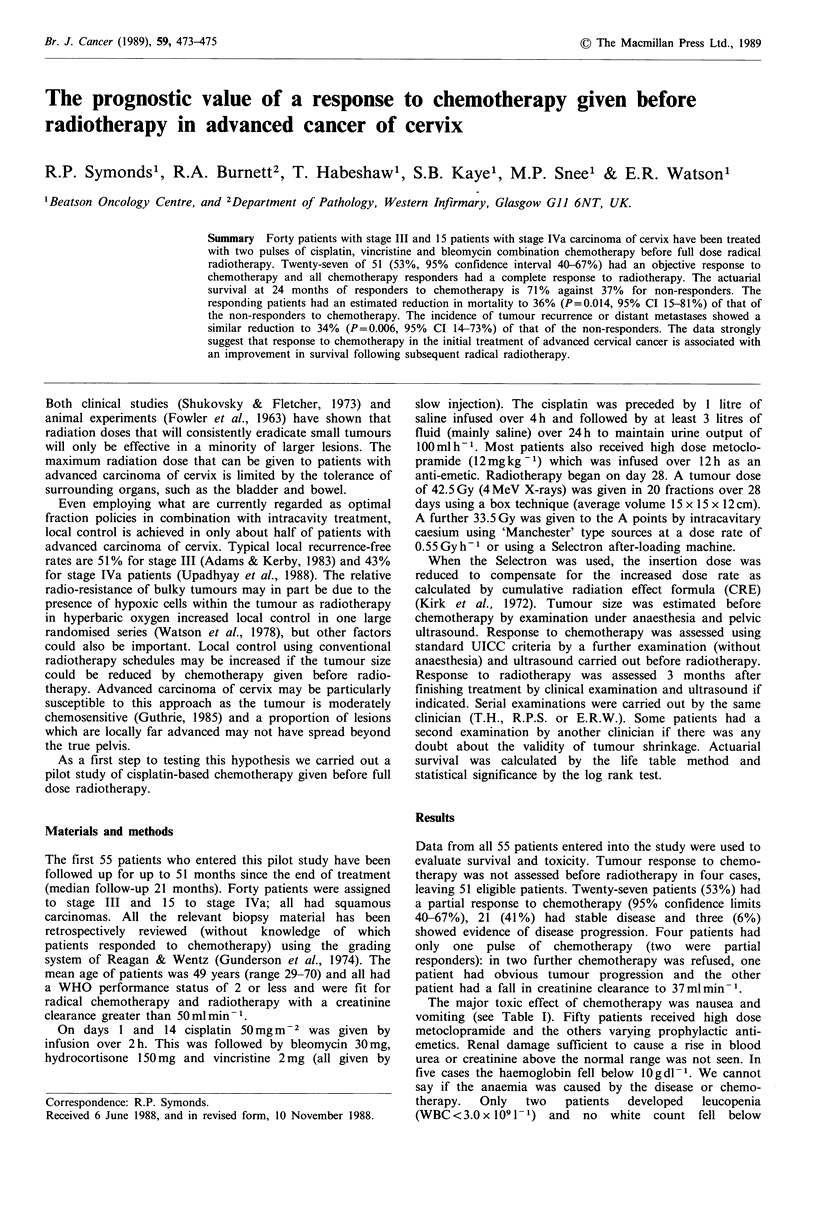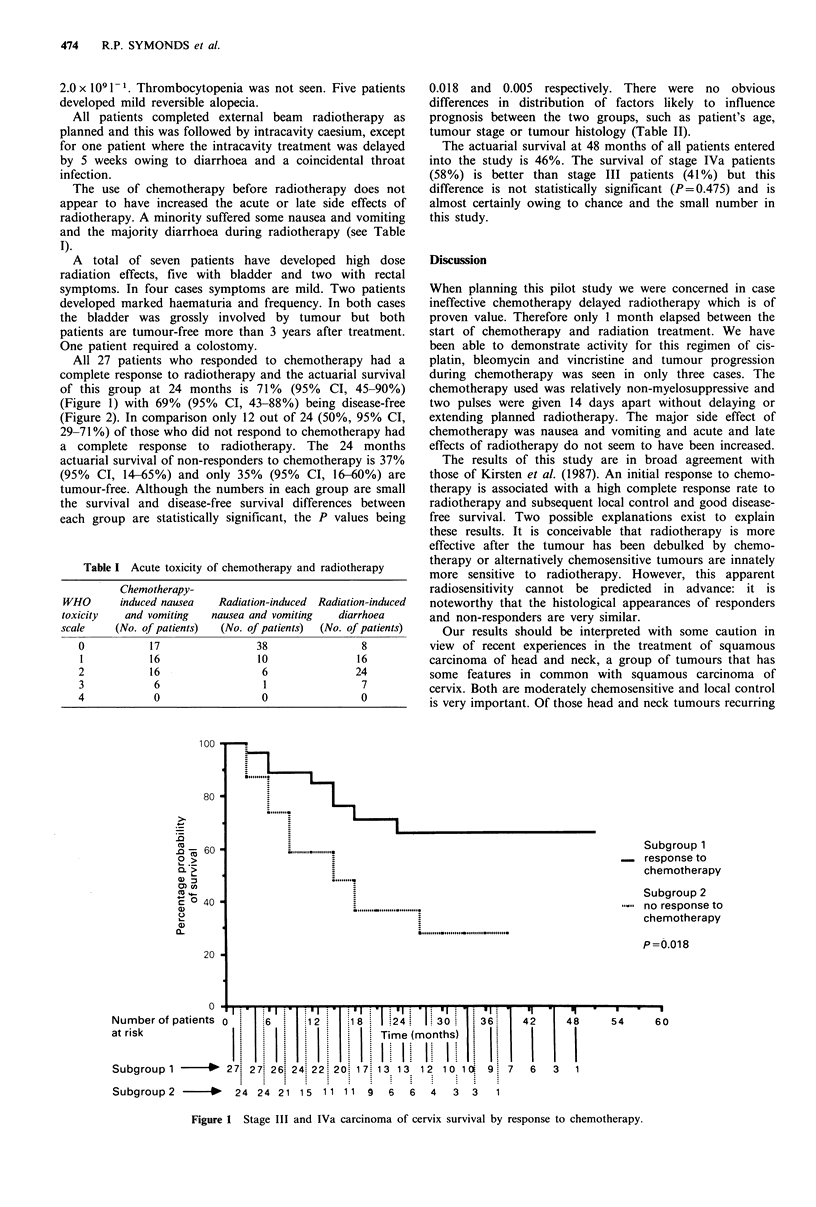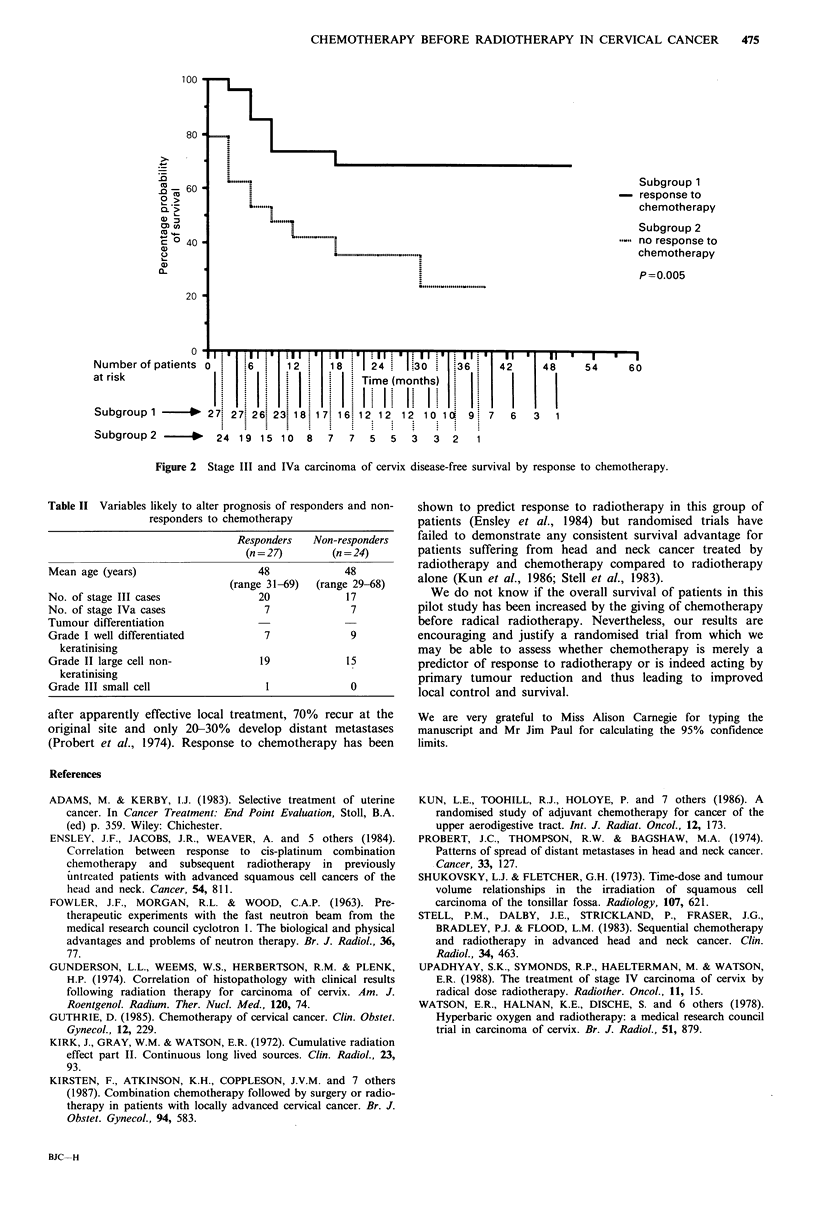# The prognostic value of a response to chemotherapy given before radiotherapy in advanced cancer of cervix.

**DOI:** 10.1038/bjc.1989.98

**Published:** 1989-03

**Authors:** R. P. Symonds, R. A. Burnett, T. Habeshaw, S. B. Kaye, M. P. Snee, E. R. Watson

**Affiliations:** Beatson Oncology Centre, Western Infirmary, Glasgow, UK.

## Abstract

Forty patients with stage III and 15 patients with stage IVa carcinoma of cervix have been treated with two pulses of cisplatin, vincristine and bleomycin combination chemotherapy before full dose radical radiotherapy. Twenty-seven of 51 (53%, 95% confidence interval 40-67%) had an objective response to chemotherapy and all chemotherapy responders had a complete response to radiotherapy. The actuarial survival at 24 months of responders to chemotherapy is 71% against 37% for non-responders. The responding patients had an estimated reduction in mortality to 36% (P = 0.014, 95% CI 15-81%) of that of the non-responders to chemotherapy. The incidence of tumour recurrence or distant metastases showed a similar reduction to 34% (P = 0.006, 95% CI 14-73%) of that of the non-responders. The data strongly suggest that response to chemotherapy in the initial treatment of advanced cervical cancer is associated with an improvement in survival following subsequent radical radiotherapy.


Br. J. Cancer (1989), 59, 473–475 © The Macmillan Press Ltd., 1989

### The prognostic value of a response to chemotherapy given before radiotherapy in advanced cancer of cervix

R.P. Symonds[1], R.A. Burnett[2], T. Habeshaw[1], S.B. Kaye[1], M.P. Snee[1] & E.R. Watson[1]

[1]*Beatson Oncology Centre, and* [2]*Department of Pathology, Western Infirmary, Glasgow G11 6NT, UK.*

**Summary** Forty patients with stage III and 15 patients with stage IVa carcinoma of cervix have been treated with two pulses of cisplatin, vincristine and bleomycin combination chemotherapy before full dose radical radiotherapy. Twenty-seven of 51 (53%, 95% confidence interval 40–67%) had an objective response to chemotherapy and all chemotherapy responders had a complete response to radiotherapy. The actuarial survival at 24 months of responders to chemotherapy is 71% against 37% for non-responders. The responding patients had an estimated reduction in mortality to 36% ($P=0.014$, 95% CI 15–81%) of that of the non-responders to chemotherapy. The incidence of tumour recurrence or distant metastases showed a similar reduction to 34% ($P=0.006$, 95% CI 14–73%) of that of the non-responders. The data strongly suggest that response to chemotherapy in the initial treatment of advanced cervical cancer is associated with an improvement in survival following subsequent radical radiotherapy.

Both clinical studies (Shukovsky & Fletcher, 1973) and animal experiments (Fowler *et al.*, 1963) have shown that radiation doses that will consistently eradicate small tumours will only be effective in a minority of larger lesions. The maximum radiation dose that can be given to patients with advanced carcinoma of cervix is limited by the tolerance of surrounding organs, such as the bladder and bowel.

Even employing what are currently regarded as optimal fraction policies in combination with intracavity treatment, local control is achieved in only about half of patients with advanced carcinoma of cervix. Typical local recurrence-free rates are 51% for stage III (Adams & Kerby, 1983) and 43% for stage IVa patients (Upadhyay *et al.*, 1988). The relative radio-resistance of bulky tumours may in part be due to the presence of hypoxic cells within the tumour as radiotherapy in hyperbaric oxygen increased local control in one large randomised series (Watson *et al.*, 1978), but other factors could also be important. Local control using conventional radiotherapy schedules may be increased if the tumour size could be reduced by chemotherapy given before radiotherapy. Advanced carcinoma of cervix may be particularly susceptible to this approach as the tumour is moderately chemosensitive (Guthrie, 1985) and a proportion of lesions which are locally far advanced may not have spread beyond the true pelvis.

As a first step to testing this hypothesis we carried out a pilot study of cisplatin-based chemotherapy given before full dose radiotherapy.

### Materials and methods

The first 55 patients who entered this pilot study have been followed up for up to 51 months since the end of treatment (median follow-up 21 months). Forty patients were assigned to stage III and 15 to stage IVa; all had squamous carcinomas. All the relevant biopsy material has been retrospectively reviewed (without knowledge of which patients responded to chemotherapy) using the grading system of Reagan & Wentz (Gunderson *et al.*, 1974). The mean age of patients was 49 years (range 29–70) and all had a WHO performance status of 2 or less and were fit for radical chemotherapy and radiotherapy with a creatinine clearance greater than 50 ml min$^{-1}$.

On days 1 and 14 cisplatin 50 mg m$^{-2}$ was given by infusion over 2 h. This was followed by bleomycin 30 mg, hydrocortisone 150 mg and vincristine 2 mg (all given by slow injection). The cisplatin was preceded by 1 litre of saline infused over 4 h and followed by at least 3 litres of fluid (mainly saline) over 24 h to maintain urine output of 100 ml h$^{-1}$. Most patients also received high dose metoclopramide (12 mg kg$^{-1}$) which was infused over 12 h as an anti-emetic. Radiotherapy began on day 28. A tumour dose of 42.5 Gy (4 MeV X-rays) was given in 20 fractions over 28 days using a box technique (average volume 15 × 15 × 12 cm). A further 33.5 Gy was given to the A points by intracavitary caesium using 'Manchester' type sources at a dose rate of 0.55 Gy h$^{-1}$ or using a Selectron after-loading machine.

When the Selectron was used, the insertion dose was reduced to compensate for the increased dose rate as calculated by cumulative radiation effect formula (CRE) (Kirk *et al.*, 1972). Tumour size was estimated before chemotherapy by examination under anaesthesia and pelvic ultrasound. Response to chemotherapy was assessed using standard UICC criteria by a further examination (without anaesthesia) and ultrasound carried out before radiotherapy. Response to radiotherapy was assessed 3 months after finishing treatment by clinical examination and ultrasound if indicated. Serial examinations were carried out by the same clinician (T.H., R.P.S. or E.R.W.). Some patients had a second examination by another clinician if there was any doubt about the validity of tumour shrinkage. Actuarial survival was calculated by the life table method and statistical significance by the log rank test.

### Results

Data from all 55 patients entered into the study were used to evaluate survival and toxicity. Tumour response to chemotherapy was not assessed before radiotherapy in four cases, leaving 51 eligible patients. Twenty-seven patients (53%) had a partial response to chemotherapy (95% confidence limits 40–67%), 21 (41%) had stable disease and three (6%) showed evidence of disease progression. Four patients had only one pulse of chemotherapy (two were partial responders): in two further chemotherapy was refused, one patient had obvious tumour progression and the other patient had a fall in creatinine clearance to 37 ml min$^{-1}$.

The major toxic effect of chemotherapy was nausea and vomiting (see Table I). Fifty patients received high dose metoclopramide and the others varying prophylactic anti-emetics. Renal damage sufficient to cause a rise in blood urea or creatinine above the normal range was not seen. In five cases the haemoglobin fell below 10 g dl$^{-1}$. We cannot say if the anaemia was caused by the disease or chemotherapy. Only two patients developed leucopenia (WBC $< 3.0 \times 10^9$ l$^{-1}$) and no white count fell below

Correspondence: R.P. Symonds.
Received 6 June 1988, and in revised form, 10 November 1988.

$2.0 \times 10^9 l^{-1}$. Thrombocytopenia was not seen. Five patients developed mild reversible alopecia.

All patients completed external beam radiotherapy as planned and this was followed by intracavity caesium, except for one patient where the intracavity treatment was delayed by 5 weeks owing to diarrhoea and a coincidental throat infection.

The use of chemotherapy before radiotherapy does not appear to have increased the acute or late side effects of radiotherapy. A minority suffered some nausea and vomiting and the majority diarrhoea during radiotherapy (see Table I).

A total of seven patients have developed high dose radiation effects, five with bladder and two with rectal symptoms. In four cases symptoms are mild. Two patients developed marked haematuria and frequency. In both cases the bladder was grossly involved by tumour but both patients are tumour-free more than 3 years after treatment. One patient required a colostomy.

All 27 patients who responded to chemotherapy had a complete response to radiotherapy and the actuarial survival of this group at 24 months is 71% (95% CI, 45–90%) (Figure 1) with 69% (95% CI, 43–88%) being disease-free (Figure 2). In comparison only 12 out of 24 (50%, 95% CI, 29–71%) of those who did not respond to chemotherapy had a complete response to radiotherapy. The 24 months actuarial survival of non-responders to chemotherapy is 37% (95% CI, 14–65%) and only 35% (95% CI, 16–60%) are tumour-free. Although the numbers in each group are small the survival and disease-free survival differences between each group are statistically significant, the $P$ values being 0.018 and 0.005 respectively. There were no obvious differences in distribution of factors likely to influence prognosis between the two groups, such as patient's age, tumour stage or tumour histology (Table II).

The actuarial survival at 48 months of all patients entered into the study is 46%. The survival of stage IVa patients (58%) is better than stage III patients (41%) but this difference is not statistically significant ($P = 0.475$) and is almost certainly owing to chance and the small number in this study.

**Table I** Acute toxicity of chemotherapy and radiotherapy

| *WHO toxicity scale* | *Chemotherapy-induced nausea and vomiting (No. of patients)* | *Radiation-induced nausea and vomiting (No. of patients)* | *Radiation-induced diarrhoea (No. of patients)* |
|---|---|---|---|
| 0 | 17 | 38 | 8 |
| 1 | 16 | 10 | 16 |
| 2 | 16 | 6 | 24 |
| 3 | 6 | 1 | 7 |
| 4 | 0 | 0 | 0 |

### Discussion

When planning this pilot study we were concerned in case ineffective chemotherapy delayed radiotherapy which is of proven value. Therefore only 1 month elapsed between the start of chemotherapy and radiation treatment. We have been able to demonstrate activity for this regimen of cisplatin, bleomycin and vincristine and tumour progression during chemotherapy was seen in only three cases. The chemotherapy used was relatively non-myelosuppressive and two pulses were given 14 days apart without delaying or extending planned radiotherapy. The major side effect of chemotherapy was nausea and vomiting and acute and late effects of radiotherapy do not seem to have been increased.

The results of this study are in broad agreement with those of Kirsten *et al.* (1987). An initial response to chemotherapy is associated with a high complete response rate to radiotherapy and subsequent local control and good disease-free survival. Two possible explanations exist to explain these results. It is conceivable that radiotherapy is more effective after the tumour has been debulked by chemotherapy or alternatively chemosensitive tumours are innately more sensitive to radiotherapy. However, this apparent radiosensitivity cannot be predicted in advance: it is noteworthy that the histological appearances of responders and non-responders are very similar.

Our results should be interpreted with some caution in view of recent experiences in the treatment of squamous carcinoma of head and neck, a group of tumours that has some features in common with squamous carcinoma of cervix. Both are moderately chemosensitive and local control is very important. Of those head and neck tumours recurring

**Figure 1** Stage III and IVa carcinoma of cervix survival by response to chemotherapy.

**Figure 2** Stage III and IVa carcinoma of cervix disease-free survival by response to chemotherapy.

**Table II** Variables likely to alter prognosis of responders and non-responders to chemotherapy

| | *Responders* ($n=27$) | *Non-responders* ($n=24$) |
|---|---|---|
| Mean age (years) | 48 (range 31–69) | 48 (range 29–68) |
| No. of stage III cases | 20 | 17 |
| No. of stage IVa cases | 7 | 7 |
| Tumour differentiation | — | — |
| Grade I well differentiated keratinising | 7 | 9 |
| Grade II large cell non-keratinising | 19 | 15 |
| Grade III small cell | 1 | 0 |

after apparently effective local treatment, 70% recur at the original site and only 20–30% develop distant metastases (Probert *et al.*, 1974). Response to chemotherapy has been shown to predict response to radiotherapy in this group of patients (Ensley *et al.*, 1984) but randomised trials have failed to demonstrate any consistent survival advantage for patients suffering from head and neck cancer treated by radiotherapy and chemotherapy compared to radiotherapy alone (Kun *et al.*, 1986; Stell *et al.*, 1983).

We do not know if the overall survival of patients in this pilot study has been increased by the giving of chemotherapy before radical radiotherapy. Nevertheless, our results are encouraging and justify a randomised trial from which we may be able to assess whether chemotherapy is merely a predictor of response to radiotherapy or is indeed acting by primary tumour reduction and thus leading to improved local control and survival.

We are very grateful to Miss Alison Carnegie for typing the manuscript and Mr Jim Paul for calculating the 95% confidence limits.

### References

ADAMS, M. & KERBY, I.J. (1983). Selective treatment of uterine cancer. In *Cancer Treatment: End Point Evaluation,* Stoll, B.A. (ed) p. 359. Wiley: Chichester.

ENSLEY, J.F., JACOBS, J.R., WEAVER, A. and 5 others (1984). Correlation between response to cis-platinum combination chemotherapy and subsequent radiotherapy in previously untreated patients with advanced squamous cell cancers of the head and neck. *Cancer,* **54,** 811.

FOWLER, J.F., MORGAN, R.L. & WOOD, C.A.P. (1963). Pre-therapeutic experiments with the fast neutron beam from the medical research council cyclotron 1. The biological and physical advantages and problems of neutron therapy. *Br. J. Radiol.,* **36,** 77.

GUNDERSON, L.L., WEEMS, W.S., HERBERTSON, R.M. & PLENK, H.P. (1974). Correlation of histopathology with clinical results following radiation therapy for carcinoma of cervix. *Am. J. Roentgenol. Radium. Ther. Nucl. Med.,* **120,** 74.

GUTHRIE, D. (1985). Chemotherapy of cervical cancer. *Clin. Obstet. Gynecol.,* **12,** 229.

KIRK, J., GRAY, W.M. & WATSON, E.R. (1972). Cumulative radiation effect part II. Continuous long lived sources. *Clin. Radiol.,* **23,** 93.

KIRSTEN, F., ATKINSON, K.H., COPPLESON, J.V.M. and 7 others (1987). Combination chemotherapy followed by surgery or radio-therapy in patients with locally advanced cervical cancer. *Br. J. Obstet. Gynecol.,* **94,** 583.

KUN, L.E., TOOHILL, R.J., HOLOYE, P. and 7 others (1986). A randomised study of adjuvant chemotherapy for cancer of the upper aerodigestive tract. *Int. J. Radiat. Oncol.,* **12,** 173.

PROBERT, J.C., THOMPSON, R.W. & BAGSHAW, M.A. (1974). Patterns of spread of distant metastases in head and neck cancer. *Cancer,* **33,** 127.

SHUKOVSKY, L.J. & FLETCHER, G.H. (1973). Time-dose and tumour volume relationships in the irradiation of squamous cell carcinoma of the tonsillar fossa. *Radiology,* **107,** 621.

STELL, P.M., DALBY, J.E., STRICKLAND, P., FRASER, J.G., BRADLEY, P.J. & FLOOD, L.M. (1983). Sequential chemotherapy and radiotherapy in advanced head and neck cancer. *Clin. Radiol.,* **34,** 463.

UPADHYAY, S.K., SYMONDS, R.P., HAELTERMAN, M. & WATSON, E.R. (1988). The treatment of stage IV carcinoma of cervix by radical dose radiotherapy. *Radiother. Oncol.,* **11,** 15.

WATSON, E.R., HALNAN, K.E., DISCHE, S. and 6 others (1978). Hyperbaric oxygen and radiotherapy: a medical research council trial in carcinoma of cervix. *Br. J. Radiol.,* **51,** 879.